# Supplementary material for: A retrospective analysis of a newly proposed imaging-etiologic classification for acute ischemic stroke with large vascular occlusion based on MRI and pathogenesis
Source: PeerJ. 2024 Oct 24;12:e18342. doi: 10.7717/peerj.18342 (PMC11512804; doi:10.7717/peerj.18342)

The table titled "peerj-103120-English_Raw_Data" provides detailed information on patients who underwent endovascular treatment (EVT) for cerebral infarction. It summarizes various clinical and surgical aspects of these cases, organized into columns as follows:

**Gender:** Indicates the gender of the patient.

**Age:** Specifies the age of the patient.

**Name of the Surgery:** Lists the specific type of endovascular treatment (EVT) performed on the patient, such as Thrombectomy with Stent Retriever+Basilar Artery Balloon Angioplasty or Arterial Thrombolysis+Thrombectomy with Stent Retriever.

**Non-Visible Cerebral Vascular Segments on MRA:** Indicates the number of non-visible cerebral vascular segments observed on Magnetic Resonance Angiography (MRA).

**Patient Subtyping in the Newly Proposed Imaging-Etiologic Classification System Based on MRI (DWI & MRA) and Medical History:** Classifies patients into subtypes using a newly proposed imaging-etiologic classification system that combines information from Magnetic Resonance Imaging (MRI) including Diffusion-Weighted Imaging (DWI) and MRA, along with medical history.

**EVT Surgery Findings Concordance with Imaging-Etiologic Classification (Y/N) ＆ Identifying the Specific Segment of Cerebral Vascular Occlusion:** Indicates whether the surgical findings during EVT concurred with the imaging-etiologic classification (Yes/No), and specifies the identified segment of cerebral vascular occlusion.

**Pathogenesis Concordance with Pre-judgment (Y/N)＆Identifying the Exact Pathogenesis:** Indicates whether the identified pathogenesis matches the pre-operative diagnosis (Yes/No) and provides details on identifying the exact pathogenesis.

**Onset Time of Cerebral Infarction（Hours）:** Records the time elapsed (in hours) from the onset of cerebral infarction to when the patient was evaluated or treated.

**Special Medical History:** Captures any special medical history of the patient that may be relevant to their condition or treatment.

The figure embedded in the Excel spreadsheet showcases the rules for registering vascular occlusion segments within the Imaging-Etiologic classification system, along with the registration protocols for non-visible cerebral vascular segments observed on MRA. In this figure, posterior circulation subtypes are denoted by Roman numerals I, II, III, and IV, which correspond to the a, b, c, and d subtypes mentioned in the article. Notably, within the etiology subtypes depicted in the figure, both types 1 and 2 are primarily attributed to LAA, thus they are uniformly registered as type 2 in the table, whereas in the article, they correspond to etiology subtype 1. Similarly, the subtype registered as 3 in the table corresponds to etiology subtype 2 in the article, and the subtype registered as 4 in the table corresponds to etiology subtype 3 in the article. (**Fig**)

**Fig:**


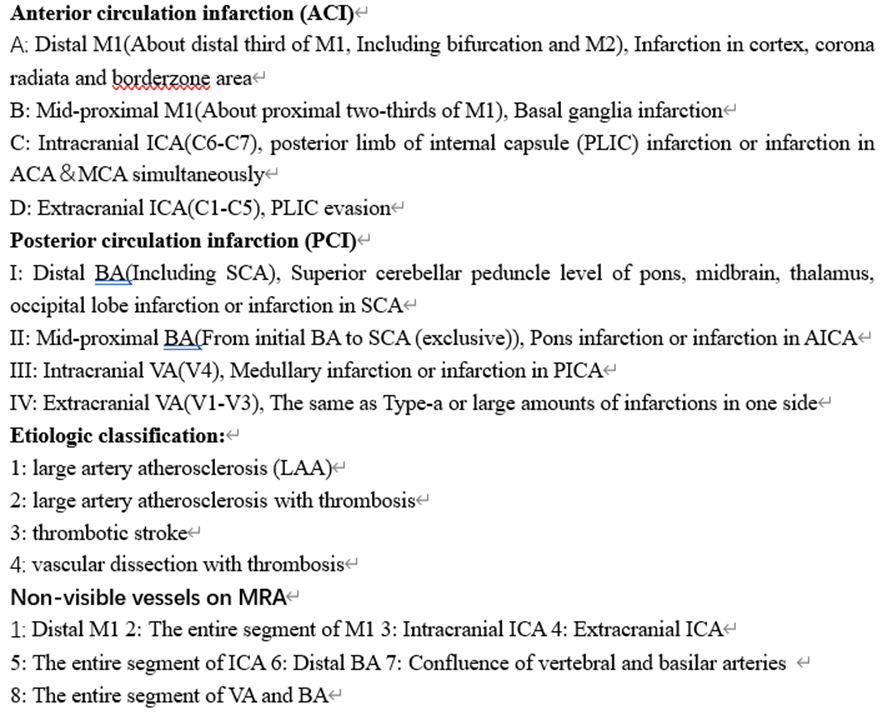

Supplement: Supplemental Information 2 [file peerj-12-18342-s002.docx]
